# Pharmacogenetic interaction between dexamethasone and *Cd36*-deficient segment of spontaneously hypertensive rat chromosome 4 affects triacylglycerol and cholesterol distribution into lipoprotein fractions

**DOI:** 10.1186/1476-511X-9-38

**Published:** 2010-04-16

**Authors:** Michaela Krupková, Lucie Šedová, František Liška, Drahomíra Křenová, Vladimír Křen, Ondřej Šeda

**Affiliations:** 1 Institute of Biology and Medical Genetics of the First Faculty of Medicine of Charles University and the General Teaching Hospital, Albertov 4, 128 00 Prague, Czech Republic; 2 Department of Metabolism and Diabetes, Institute for Clinical and Experimental Medicine, Vídeňská 1958/9, 140 21 Prague, Czech Republic; 3 Institute of Physiology and Center for Applied Genomics, Academy of Sciences of the Czech Republic, Videnska 1083, 142 20 Prague, Czech Republic; 4 Centre de Recherche, Centre hospitalier de l'Université de Montreal (CRCHUM), Technopole Angus, 2901 Rachel E., H1W4A4 Montreal (Quebec), Canada

## Abstract

Dexamethasone (DEX) is known to induce diabetes and dyslipidemia. We have compared fasting triacylglycerol and cholesterol concentrations across 20 lipoprotein fractions and glucose tolerance in control (standard diet) and DEX-treated 7-month-old males of two rat strains, Brown Norway (BN) and congenic BN.SHR-(*Il6*-*Cd36*)/Cub (BN.SHR4). These two inbred strains differ in a defined segment of chromosome 4, originally transferred from the spontaneously hypertensive rat (SHR) including the mutant *Cd36 *gene, a known target of DEX. Compared to BN, the standard-diet-fed BN.SHR4 showed higher cholesterol and triacylglycerol concentrations across many lipoprotein fractions, particularly in small VLDL and LDL particles. Total cholesterol was decreased by DEX by more than 21% in BN.SHR4 contrasting with the tendency to increase in BN (strain*DEX interaction p = 0.0017). Similar pattern was observed for triacylglycerol concentrations in LDL. The LDL particle size was significantly reduced by DEX in both strains. Also, while control BN and BN.SHR4 displayed comparable glycaemic profiles during oral glucose tolerance test, we observed a markedly blunted DEX induction of glucose intolerance in BN.SHR4 compared to BN. In summary, we report a pharmacogenetic interaction between limited genomic segment with mutated *Cd36 *gene and dexamethasone-induced glucose intolerance and triacylglycerol and cholesterol redistribution into lipoprotein fractions.

## Findings

Glucocorticoids (GC) have been utilized for decades in treatment of wide variety of inflammatory, allergic, hematological and other disorders. In spite of their demonstrated therapeutic value, glucocorticoid treatment is often accompanied with substantial side-effects, including dyslipidemia, diabetes, obesity, osteoporosis, muscle wasting, impaired wound healing or rheumatoid arthritis [1]. While the molecular mechanisms of the GC-induced metabolic disturbances have been subjected to intensive investigation [2], the genetic basis of the interindividual differences in response to GC received only limited attention so far. Several genes have been proposed to transduce or modulate the metabolic effects of glucocorticoids, including functional candidates like glucocorticoid receptor [3], 11β-hydroxysteroid dehydrogenases 1 and 2 (11β-HSD1, 2) [4] and corticosteroid-binding globulin (CBG) [5], and peroxisome proliferator-activated receptor alpha (PPARα) [6]. We have previously reported a comprehensive set of quantitative trait loci related to genomic architecture of metabolic syndrome including its dynamics in response to dexamethasone (DEX)-induced derangements of lipid and carbohydrate metabolism [7].

In the current study, we tested the effect of deficiency of one of the DEX-target genes, fatty acid translocase *Cd36 *[8,9], on the DEX-induced metabolic changes. To that end, we have compared triacylglycerol and cholesterol concentrations across 20 lipoprotein fractions and glucose tolerance in control and DEX-treated adult males of two rat strains, Brown Norway (BN) and congenic BN.SHR-(*Il6*-*Cd36*)/Cub (BN.SHR4 hereafter; Rat Genome Database [10] (RGD) ID 728142). These two inbred strains differ in a defined segment of chromosome 4, originally transferred from the spontaneously hypertensive rat (SHR) including the mutant *Cd36 *gene into the genomic background of BN to create BN.SHR4 [11,12].

All experiments were performed in agreement with the Animal Protection Law of the Czech Republic (311/1997) which is in compliance with the European Community Council recommendations for the use of laboratory animals 86/609/ECC and were approved by the Ethical committee of the First Faculty of Medicine. Animals were held under temperature and humidity controlled conditions on 12 h/12 h light-dark cycle. At all times, the animals had free access to food and water. Male BN (n = 12) and BN.SHR4 (n = 13) rats were fed standard laboratory chow *ad libitum*. At the age of 7 months, the rats were randomly split into control (n = 6 and 7 for BN and BN.SHR4, respectively) and experimental groups (n = 6/strain). Experimental groups were administered dexamethasone (Dexamed, Medochemie) in drinking water (2.6 μg/ml) for three days as described previously [7]. The OGTT was performed after overnight fasting. Blood for glycaemia determination (Ascensia Elite Blood Glucose Meter; Bayer HealthCare, Mishawaka, IN, validated by Institute of Clinical Biochemistry and Laboratory Diagnostics of the First Faculty of Medicine) was drawn from the tail at intervals of 0, 30, 60, 120 and 180 minutes after the intragastric glucose administration to conscious rats (3 g/kg body weight, 30% aqueous solution). Plasma lipoproteins were analyzed by an on-line dual enzymatic method for simultaneous quantification of cholesterol, triacylglycerol and free glycerol by HPLC at Skylight Biotech Inc. (Akita, Japan) according to the procedure described previously [13].

The control groups of both strains showed similar morphometric profile, BN.SHR4 had slightly lower relative heart and testes weights compared to BN. DEX-treated BN.SHR4 displayed greater body weight loss while maintaining food intake comparable to BN (Table 1). Despite that, the reduction of retroperitoneal fat mass was more pronounced in BN (Table 1).

**Table 1:** Morphometric comparison of BN vs. BN.SHR4 rats.

	CONTROL		DEXAMETHASONE	
Trait	BN (n = 6)	BN.SHR4 (n = 7)	BN (n = 6)	BN.SHR4 (n = 6)
Body weight (b.wt.), g	281 ± 9	312 ± 15	257 ± 7	262 ± 10†
Liver wt, g/100 g b.wt.	2.17 ± 0.03	2.15 ± 0.03	2.40 ± 0.03‡	2.27 ± 0.03^a^,*
Heart wt, g/100 g b.wt.	0.31 ± 0.01	0.28 ± 0.01^a^	0.34 ± 0.01*	0.34 ± 0.01‡
Kidney wt, g/100 g b.wt.	0.55 ± 0.01	0.52 ± 0.01	0.58 ± 0.02	0.58 ± 0.01‡
Adrenals wt, mg/100 g b.wt.	15.2 ± 1.7	13.6 ± 0.6	12.9 ± 0.6	12.3 ± 1.3
Testes wt, g/100 g b.wt	1.09 ± 0.03	0.97 ± 0.04^a^	1.13 ± 0.03	1.03 ± 0.03
EFP wt, g/100 g b.wt.	0.80 ± 0.03	0.89 ± 0.05	0.73 ± 0.03	0.82 ± 0.02
RFP wt, g/100 g b.wt.	0.36 ± 0.03	0.40 ± 0.04	0.25 ± 0.02*	0.34 ± 0.02^a^

The significance levels are indicated as follows: a...p < 0.05, respectively for differences between BN and BN.SHR4 under conditions of a single diet; *, †, ‡... p < 0.05, 0.01 and 0.001, respectively, for DEX effect within individual strain. Values are shown as mean ± S.E.M.; b.wt....body weight; EFP...epididymal fat pad; RFP...retroperitoneal fat pad.

**Figure 1:**
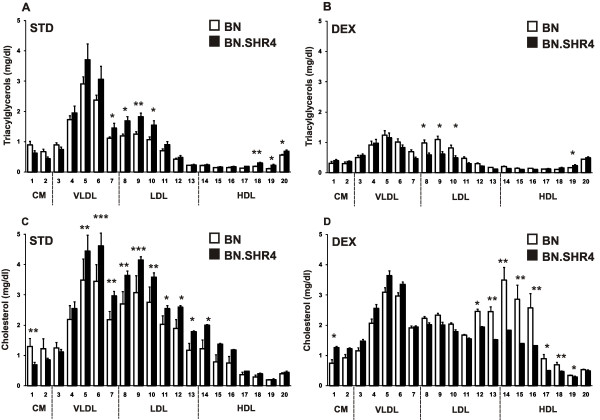
**Triacylglycerol and cholesterol profile of BN vs. BN.SHR4**. The triacylglycerol (A, B) and cholesterol (C, D) content in 20 lipoprotein subfractions in standard diet-fed (STD, A and C) and dexamethasone-treated (DEX, B and D) BN (open symbols) vs. BN.SHR4 (closed symbols) male rats (n = 6/strain*treatment). Within the graph, the significance levels of strain comparison (BN vs. BN.SHR4, two-way ANOVA with STRAIN and DEX as major factors followed by post-hoc Tukey's honest significance difference test) are indicated as follows: *...p < 0.05; **...p < 0.01; ***...p < 0.001. The allocation of individual lipoprotein subfractions to major lipoprotein classes is shown in order of particle's decreasing size from left to right. CM...chylomicron, VLDL...very low-density lipoprotein, LDL...low density lipoprotein, HDL...high-density lipoprotein.

Although total serum triacylglycerols (TG) were not significantly different between the control groups of the two strains, in-depth analysis revealed TG elevation in BN.SHR4 in small very low-density lipoprotein (VLDL), large, medium and small low-density lipoprotein (LDL) and small and very small high-density lipoprotein (HDL) subfractions (Figure 1A). DEX induced substantially more robust decreases of TG in BN.SHR4 except for small HDL. Therefore, DEX-treated BN had higher concentrations of TG in large, medium and small LDL (Figure 1B). There were no strain- or DEX-related differences in fasting glycerol levels (data not shown).

Standard diet-fed BN.SHR4 showed in comparison to BN higher cholesterol content in most lipoprotein fractions except chylomicrons (Figure 1C). Total cholesterol was decreased by DEX by more than 21% in BN.SHR4 contrasting with the tendency to increase in BN (strain*DEX interaction p = 0.0017, Table 2). When analyzed in detail, DEX-treated BN displayed higher cholesterol concentrations in very small LDL and across HDL spectrum (Figure 1D). Concomitantly, the HDL particle size increased only in BN (Table 3).

**Table 2:** Two-way analysis of variance (ANOVA) results.

Phenotype	STRAIN	DEX	STRAIN*DEX
Body weight (b.wt.)	*0.11*	**0.0027**	*0.25*
Liver, g/100 g b.wt.	*0.08*	**0.0004**	*0.07*
Heart, g/100 g b.wt.	*0.09*	**0.0005**	*0.48*
Kidney, g/100 g b.wt.	*0.41*	**0.0007**	*0.24*
Adrenals, mg/100 g b.wt.	*0.37*	*0.15*	*0.71*
EFP wt., g/100 g b.wt.	**0.012**	*0.06*	*0.78*
RFP wt., g/100 g b.wt.	*0.07*	**0.0023**	*0.26*
Testes wt., g/100 g b.wt.	**0.0027**	*0.16*	*0.67*
Glucose (0 min)	*0.20*	**<0.0001**	*0.50*
Glucose (30 min)	*0.31*	**<0.0001**	*0.14*
Glucose (60 min)	**0.0015**	**<0.0001**	**0.0005**
Glucose (120 min)	**0.0043**	**0.0001**	**0.046**
Glucose (180 min)	*0.07*	**<0.0001**	**0.032**
AUC OGTT (0-180 min)	**0.010**	**<0.0001**	**0.0048**
Glycerol	*0.37*	*0.11*	*0.38*
			
**Triacylglycerol (TG)**			
**Total TG**	*0.21*	**0.0034**	*0.74*
Chylomicron TG	*0.60*	*0.48*	*0.08*
VLDL-TG	*0.19*	**0.0026**	*0.99*
LDL-TG	*0.37*	**0.0002**	**0.017**
HDL-TG	**0.028**	*0.14*	*0.59*
F1 (CM)	*0.54*	*0.43*	*0.09*
F2 (CM)	*0.69*	*0.57*	*0.06*
F3 (large VLDL)	*0.44*	*0.62*	*0.13*
F4 (large VLDL)	*0.19*	*0.09*	*0.50*
F5 (large VLDL)	*0.15*	**0.0011**	*0.83*
F6 (medium VLDL)	*0.18*	**<0.0001**	*0.42*
F7 (small VLDL)	*0.28*	**<0.0001**	*0.11*
F8 (large LDL)	*0.32*	**0.0004**	**0.0014**
F9 (medium LDL)	*0.36*	**0.0002**	**0.0031**
F10 (small LDL)	*0.29*	**0.0002**	**0.013**
F11 (very small LDL)	*0.52*	**0.0003**	*0.11*
F12 (very small LDL)	*0.70*	**0.0060**	*0.45*
F13 (very small LDL)	*0.95*	*0.06*	*0.76*
F14 (very large HDL)	*0.99*	*0.33*	*0.60*
F15 (very large HDL)	*0.81*	*0.66*	*0.48*
F16 (large HDL)	*0.62*	*0.39*	*0.57*
F17 (medium HDL)	**0.021**	*0.44*	*0.81*
F18 (small HDL)	**0.0021**	**0.0023**	*0.46*
F19 (very small HDL)	**0.0023**	*0.10*	*0.93*
F20 (very small HDL)	**0.015**	**0.0051**	*0.74*
**Cholesterol (C)**			
**Total C**	*0.26*	*0.28*	**0.0017**
Chylomicron C	*0.58*	*0.89*	**0.0091**
VLDL-C	**0.0018**	**0.0081**	*0.35*
LDL-C	*0.13*	**0.0002**	**0.0006**
HDL-C	*0.21*	**0.0036**	**0.0059**
			
F1 (CM)	*0.55*	*0.82*	**0.0013**
F2 (CM)	*0.32*	*0.97*	*0.06*
F3 (large VLDL)	*0.54*	*0.38*	*0.10*
F4 (large VLDL)	**0.028**	*0.75*	*0.69*
F5 (large VLDL)	**0.0026**	**0.016**	*0.40*
F6 (medium VLDL)	**0.0009**	**0.0005**	*0.08*
F7 (small VLDL)	**0.025**	**0.0017**	**0.045**
F8 (large LDL)	*0.06*	**<0.0001**	**0.0085**
F9 (medium LDL)	**0.030**	**<0.0001**	**0.0007**
F10 (small LDL)	*0.08*	**<0.0001**	**0.0059**
F11 (very small LDL)	*0.11*	**0.0001**	**0.032**
F12 (very small LDL)	*0.52*	*0.94*	**0.0038**
F13 (very small LDL)	*0.47*	**0.014**	**0.0009**
F14 (very large HDL)	*0.21*	**0.0050**	**0.0019**
F15 (very large HDL)	*0.20*	**0.0048**	**0.0056**
F16 (large HDL)	*0.22*	**0.0067**	**0.018**
F17 (medium HDL)	*0.15*	**0.089**	**0.015**
F18 (small HDL)	*0.30*	**0.0004**	**0.0096**
F19 (very small HDL)	*0.41*	**<0.0001**	*0.11*
F20 (very small HDL)	*0.87*	**<0.0001**	**0.024**
			
**Lipoprotein particle size**			
VLDL-TG	*0.06*	**<0.0001**	**<0.0001**
LDL-C	*0.15*	**<0.0001**	*0.09*
HDL-C	*0.59*	*0.44*	**0.0038**

The significance levels of two-way ANOVA's STRAIN, DEX and STRAIN*DEX factor interactions are shown (significant p values in bold, non-significant in italics). For glucose tolerance test, the time in minutes after the oral glucose load is indicated in parentheses. b.wt....body weight; EFP...epididymal fat pad; RFP...retroperitoneal fat pad. AUC OGTT...area under the glycaemic curve of the oral glucose tolerance test.

**Table 3:** Lipoprotein particle size comparison between BN and BN.SHR4.

	CONTROL		DEXAMETHASONE	
Trait (nm)	BN (n = 6)	BN.SHR4 (n = 7)	BN (n = 6)	BN.SHR4 (n = 6)
VLDL	44.51 ± 0.09	43.55 ± 0.17^b^	44.68 ± 0.28	46.60 ± 0.32‡,^c^
LDL	23.18 ± 0.07	23.15 ± 0.17	22.11 ± 0.06‡	22.54 ± 0.18†,^c^
HDL	12.36 ± 0.07	12.64 ± 0.09	12.78 ± 0.05†	12.38 ± 0.17^a^

The significance levels are indicated as follows: a,b,c...p < 0.05, 0.01 and 0.001, respectively for differences between BN and BN.SHR4 under conditions of a single diet; *, †, ‡... p < 0.05, 0.01 and 0.001, respectively, for DEX effect within individual strain. Values are shown as mean ± S.E.M.

**Figure 2:**
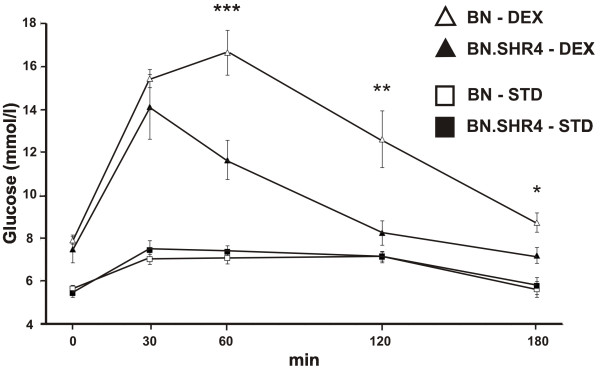
**Oral glucose tolerance test of BN vs. BN.SHR4**. Oral glucose tolerance test (OGTT) in control (squares) and dexamethasone-treated (DEX; triangles) male BN (open symbols) and BN.SHR4 (closed symbols) male rats. Within the graphs, the significance levels of strain comparison (BN vs. BN.SHR4) by post-hoc Tukey's honest significance difference test of the two-way ANOVA with STRAIN and DEX as major factors (STATISTICA 8 CZ) are indicated as follows: *...p < 0.05; **...p < 0.01; ***...p < 0.001.

While there was no strain difference in response to glucose bolus administration in the control groups, we observed a markedly diminished DEX induction of glucose intolerance in BN.SHR4 compared to BN (Figure 2, reflected by strain*DEX interaction in two-way ANOVA, p = 0.005). Actually, the incremental area under the glycaemic curve failed to increase significantly in BN.SHR4 (244 ± 34 vs. 418 ± 66 mmol/l/180 min in control vs. DEX-treated animals, respectively, p = 0.23), while we observed more than threefold, significant increase in BN rats (222 ± 30 vs. 817 ± 110 mmol/l/180 min in control vs. DEX-treated animals, respectively, p = 0.0002).

Our study presents a pharmacogenetic interaction between limited genomic segment with mutated *Cd36 *gene and dexamethasone-induced glucose intolerance and triacylglycerol and cholesterol redistribution into lipoprotein fractions. Genetic variation in fatty acid translocase *CD36 *has been previously linked with dyslipidemia and insulin resistance both in experimental models [14,15] and in human subjects [16,17]. Moreover, we have established *Cd36 *as key determinant of the metabolic effects of insulin-sensitizer drugs - thiazolidinediones by demonstrating their blunted action both in SHR [18] and BN.SHR4 [12,19]. The BN.SHR4 displays several derangements of lipid and carbohydrate metabolism compared to BN while fed standard or high-sucrose diet [11]. In this study, the *Cd36*-deficient congenic showed reduced susceptibility to diabetogenic action of DEX and even partial improvement of its lipid profile, contrasting with its BN progenitor. Dexamethasone is known to induce whole body insulin resistance and affect lipid metabolism after both short and long-term administration [20,1,21] while CD36 is one of its target genes [8,9]. We have previously shown DEX to concomitantly induce both muscle-specific insulin resistance and dyslipidemia in experimental models of metabolic syndrome including spontaneously hypertensive rat-derived congenic strain [22], polydactylous rat as well as BN [7]. The distinct pattern reported in the current study, i.e. induction of glucose intolerance by DEX combined with tendency to reduce concentrations of triacylglycerol and cholesterol in certain lipoprotein fractions may be attributed to short term administration of one-tenth of the dose used in our prior studies [7,22]. One of the limitations of the current study is the possibility that other genes apart from mutated *Cd36 *present in the differential segment might be involved in the underlying mechanism of distinct metabolic response of the two strains, this issue will be addressed in future studies by e.g. generating *Cd36 *knockout rats [23]. Although it is premature to speculate on the detailed mechanism of the observed interaction, which might involve enhanced glucose utilization in peripheral tissues due to ineffective fatty-acid uptake [18], we may hypothesize that *Cd36 *and/or some other gene(s) present in the chromosome 4 differential segment may represent pharmacogenetic hubs [24] of particular importance for metabolic actions of glucocorticoids.

## Competing interests

The authors declare that they have no competing interests.

## Authors' contributions

MK and LS carried out the metabolic component of the study and drafted the manuscript. FL and DK participated in the design of the study and performed the statistical analysis. OS and VK conceived the study, and participated in its design and coordination and helped to draft the manuscript. All authors read and approved the final manuscript.
